# Mutation Analysis of Exon 1 in the Hemoglobin Subunit Beta (HBB) Gene in Beta-Thalassemia

**DOI:** 10.7759/cureus.65198

**Published:** 2024-07-23

**Authors:** K Sharath Kumar, Mallanagouda M Patil, Rudragouda Bulagouda, Gurushantappa S Kadakol

**Affiliations:** 1 Department of Paediatrics, BLDE (Deemed to be University) Shri B. M. Patil Medical College, Hospital, and Research Centre, Vijayapura, IND; 2 Department of Anatomy, BLDE (Deemed to be University) Shri B. M. Patil Medical College, Hospital, and Research Centre, Vijayapura, IND; 3 Genetics Laboratory, Department of Anatomy, BLDE (Deemed to be University) Shri B. M. Patil Medical College, Hospital, and Research Centre, Vijayapura, IND

**Keywords:** beta-thalassemia, sanger sequencing, pcr, exon 1, hbb gene

## Abstract

Introduction

Thalassemia is a widely prevalent monogenic hematological disorder found worldwide. It exists in two forms: alpha- and beta-thalassemia. Alterations in the hemoglobin subunit beta (HBB) gene cause beta-thalassemia, with missense and point mutations affecting beta-globin synthesis. Consequently, genetic screening for beta-thalassemia is essential for genetic counseling, carrier screening, and prenatal diagnosis.

Aim and objective

This study aims to examine and identify mutations in the exon 1 region of the HBB gene in beta-thalassemia patients from the Vijayapura region.

Methods

This study involved 47 clinically diagnosed children with beta-thalassemia from a hospital in Vijayapura, India. Detailed clinical histories of all patients were recorded. Genomic DNA was extracted from the blood samples of these patients and subjected to polymerase chain reaction (PCR) using exon-specific primers for the HBB gene. The PCR products were then sequenced using the capillary-based Sanger sequencing method to identify mutations in the HBB gene.

Results

A total of 47 clinically diagnosed beta-thalassemia patients were included in the study, comprising 30 males and 17 females, aged between one and 20 years. Sequencing analysis of exon 1 in the beta-globin gene identified 17 beta-thalassemia variants. The most common mutation observed was T>G, G>C, C>A, and C>T in the exon 1 region of the HBB gene.

Conclusion

This study identifies the pattern of beta-thalassemia mutations, aiding in the prevention of the disorder through prenatal diagnosis and genetic counseling. Mutations can alter codon sequences, affecting protein production. Research highlights the importance of a primary prevention program to analyze mutations and sequence variations at the molecular level, thereby helping to address numerous genetic disorders.

## Introduction

Thalassemia is one of the most common monogenic disorders globally, with about 100,000 children dependent on transfusions for the disease. In India, the prevalence rate is significant, with 8,000-10,000 children born annually with thalassemia-related conditions, representing 10% of the global burden [1]. The carrier frequency of thalassemia in India ranges from 5% to 17%, varying across different states and geographical regions [2]. This disorder is highly prevalent in Asia, Africa, the Mediterranean region, and particularly malaria-endemic areas [3].

Beta-thalassemia, an autosomal recessive disorder, involves structural variations in the beta-globin gene, resulting from a deficiency of beta-globin chains [4]. Over 300 different mutations in the beta-globin gene have been identified. The hemoglobin subunit beta (HBB) gene encodes the beta-globin chains of hemoglobin, which, along with alpha chains, form the protein that carries oxygen in red blood cells. This gene, comprising three exons and 10,106 base pairs of linear DNA, plays a crucial role in the pathogenesis of beta-thalassemia. Mutations in the HBB gene alter the beta-globin chain, leading to significant structural changes in the protein. Beta-thalassemia is characterized by decreased or absent beta-globin synthesis due to point mutations [5]. Research has shown that Indian subpopulations exhibit extensive heterogeneity in beta-thalassemia mutations [6]. However, data on HBB gene mutations, particularly in Southern India, remains limited. This study aims to investigate beta-thalassemia mutations in exon 1 of the HBB gene in these populations.

## Materials and methods

Patient selection

The study enrolled patients from the Department of Paediatrics of BLDE (Deemed to be University) Shri B. M. Patil Medical College, Hospital, and Research Centre in Vijayapura, Karnataka, India. The research was conducted in the Genetics Laboratory and Centre for Advanced Medical Research (CAMR) between January 2021 and June 2022. An Institutional Ethical Certificate (approval number: IEC/09/2021) dated January 22, 2021, was obtained from the Institutional Ethical Committee of BLDE (Deemed to be University). Before collecting blood samples, the study's purpose and procedures were thoroughly explained to the patients and their families, ensuring informed consent. A total of 47 patients, from both rural and urban areas of North Karnataka, participated in the study, selected using a structured questionnaire.

Inclusion criteria

The study included children clinically diagnosed with beta-thalassemia, aged between six months and 18 years, who were registered in the Department of Paediatrics of Shri B. M. Patil Medical College, Hospital, and Research Centre.

Exclusion criteria

Patients and their families who did not consent to provide blood samples were excluded. Additionally, other hemoglobinopathies were not included in this study.

Sample size calculation

In the present study with 47 samples, the anticipated proportion of mutations seen in beta-thalassemia patients was 8% [7], with a 95% level of confidence and a 10% absolute precision. The sample size calculation formula used was n=z2 p*qd2 where z is the Z statistic at the α level of significance, d2 is the absolute error, p is the proportion rate, and q is 100-p.

Clinical sample collection

A total of 1 ml of blood samples were collected from children with beta-thalassemia major using K2 EDTA vacutainers (LabTech Disposables). These samples were obtained from both the inpatient department (IPD) and outpatient department (OPD) of the Department of Paediatrics. After collection, the samples were processed in the laboratory following standard procedures and stored at 4°C for further research.

Extraction of DNA from blood

The DNA extraction from blood involved using approximately 200 μl of blood samples with a DNA isolation kit (Qiagen Kit, Germany). The quality and quantity of DNA samples were assessed using a multimode reader (Teckon). Primers were designed according to the gene of interest, covering the full length of exon 1 (reference sequence: NG_059281). The primer design utilized various bioinformatics tools such as Genome Build 36 and UCSC In-Silico Polymerase Chain Reaction (PCR). The designed primers were synthesized by Oligo Synthesizer Laboratory (Eurofins, India). Details of the primer sequences and annealing temperatures for the HBB gene are presented in Table 1.

**Table 1:** Primer sequences and annealing temperatures used in exon 1 in the HBB gene HBB: hemoglobin subunit beta

Name of the primer	Sequence	Amplicon size	Annealing temperature
Th1F	AGGGTTGGCCAATCTACTCC	287	60°C
Th 1R	GTCTCCACATGCCCAGTTTC		

Molecular analysis

The multiple copies of DNA were obtained by PCR in a total reaction volume of 20 μl, comprising 1 μl of DNA (20-100 ng/μl), 0.4 μl each of forward and reverse primers (5 pmol), 10 μl of ready-made master mix (Takara) containing Taq buffer, MgCl2, dNTPs, and Taq polymerase enzyme, and 7.2 μl of nuclease-free water. PCR was conducted using a thermocycler (Thermo Make Virit, USA) under the following cyclic conditions: initial denaturation at 95°C for five minutes, followed by 35 cycles of denaturation at 95°C for 30 seconds, primer annealing at 60°C for 45 seconds, primer extension at 72°C for one minute, and a final extension at 72°C for five minutes. Confirmation of PCR products was achieved using 2% agarose gel electrophoresis with a 100 bp ladder (4 μl solution).

Following confirmation, all PCR products underwent automated DNA sequencing (ABI_3500xl) using both forward and reverse primers for each sample. Electropherograms obtained from automated DNA sequencing were analyzed for sequence quality using Sequence Analysis Software (ABI). Sequence alignment was performed using Variant Reporter Software (ABI).

## Results

The study comprised 47 participants clinically diagnosed with beta-thalassemia. There were 30 males and 17 females, with patients' ages ranging from six months to 20 years, with the majority being children under five years, accounting for 51%. Details of the age distribution of patients are provided in Table 2.

**Table 2:** Age distribution of beta-thalassemia patients

Age (years)	No. of children	n (%)
≤5	24	51
6-10	14	29
≥11	12	25
Total	47	100

In the study, researchers observed a consanguineous history in 29 thalassemia children (61.7%), while the remainder did not have a consanguineous history. The status of consanguinity is detailed in Table 3.

**Table 3:** Consanguineous status (n=47)

Consanguineous history	No. of patients	n (%)
Yes	29	61.70
No	18	38.2
Total	47	100

Most of the children did not exhibit splenomegaly, accounting for 53%. Splenectomy was performed in seven children (15%), and severe splenomegaly was observed in four children (8.5%). Grades of splenic enlargement are presented in Table 4.

**Table 4:** Grades of splenic enlargement (n=47)

Spleen	No. of children	n (%)
No	25	53
Mild	3	6.5
Moderate	8	17
Severe	4	8.5
Splenectomy	7	15
Total	47	100

All sequences were analyzed using Sequence Analysis Software (ABI). Out of 47 thalassemia children, missense and synonymous mutations were observed in 17 samples, with transversions (T>A) being the most common, followed by transitions. In the remaining samples, no mutations were found. However, the researchers evaluated the study results. Among the 17 thalassemia children with mutations in their HBB gene, 10 were males and seven were females. Details of beta-thalassemia mutations are outlined in Table 5. Consanguineous history was present in 11 children, seven patients exhibited massive splenomegaly, and splenectomy was performed on two children. Pretransfusion Hb levels were less than 5 gm/dl in all children. Hepatomegaly was observed in all children. Ejection systolic murmur was noted in two children, and stunting was observed in seven children. All children exhibited a hemolytic facial appearance and were undergoing iron chelation therapy.

**Table 5:** Beta-thalassemia mutations observed in our study

Sl. no.	Sample no.	Nucleotide change	HGVS code	Unique identification code	Amino acid change	Variant
1	1	T>A	HBB:c.162T>A	rs63751103	p.F72L	Missense
2	2	T>G	HBB:c.215T>G	rs1554917888	p.F72C	Missense
3	6	T>A	HBB:c.177T>A	rs63751103	p.P59=	Synonymous
4	7	T>A	HBB:c.111T>A	rs63750532	p.V19P	Missense
5	9	T>G	HBB:c.216G>A	rs1554917888	p.F72L	Missense
6	10	G>C	HBB:c.187G>C	rs34933455	p.A63P	Missense
7	12	T>A	HBB:c.182T>A	rs33931779	p.V61D	Missense
8	16	C>A	HBB:c.41C>A	rs35203747	p.A14D	Missense
9	17	G>C	HBB:c.193G>C	rs36107977	p.G65R	Missense
10	18	G>C	HBB:c.194G>C	rs36107977	p.G65D	Missense
11	19	T>A	HBB:c.104T>A	rs1135101	p.V35D	Missense
12	20	T>A	HBB:c.182T>A	rs3393177	p.V61D	Missense
13	24	T>G	HBB:c.164T>G	rs281864583	p.V55G	Missense
14	25	T>A	HBB:c.164T>A	rs281864583	p.V55D	Missense
15	26	T>G	HBB:c.167T>G	rs1564875331	p.M56R	Missense
16	31	G>C	HBB:c.198G>C	rs35747961	p.K66N	Missense
17	32	111T>A	HBB:c.111T>A	rs63750532	p.P37=	Missense

## Discussion

The exon 1 region of the HBB gene in children of our region exhibited common mutations. The study identified mutations in 17 thalassemia children out of a total of 47. Missense mutations predominated, with transversions being more frequent than transitions. The most prevalent mutation type observed was T>A, particularly at positions 177, 164, and 111 in most sequenced samples. T>G mutations were observed at positions 215 and 216, while C>A and C>T variants occurred at low frequencies. T>A mutations were evenly distributed across all subjects analyzed.

In this study, T>A missense mutations were more frequent compared to G>C mutations found in other studies. In beta-thalassemia, the most common mutations included G→C and G→T changes at IVS 1-1, IVS 1-5, codons 8/9, and codons 41/42 (TCTT) and a 619 bp deletion. IVS-I-5 (G→C) mutations were notably prevalent in India, with a high occurrence observed in the Sindhi and Lohana communities in Gujarat. Rare beta-thalassemia mutations, including mild β++ promoter mutation -88 (C-T) in Punjab, were also noted [8-10].

The study reported T>A missense mutations in eight patients. Another study on beta-thalassemia variants identified prevalent mutations such as IVS-I-5 G>C, IVS-II-16 G>C, IVS-II-74 T>G, and mutations affecting codon 3 and the Poly A site [11,12]. Notably, the present study did not investigate intronic variants, which is a limitation.

Research by Borah et al.'s group highlighted six mutations accounting for 90-94% of beta-thalassemia mutations in Indian populations, including a 619 bp deletion and mutations in codons 8/9, codons 41/42, IVS 1-1 (G-T), and IVS 1-5 (G->C). The study observed T>A missense mutations predominantly at positions 111, 164, and 177 in most sequenced samples, with T>G mutations at positions 215 and 216 [13]. In the eastern part of India, IVS 1-5 (G>C) was the most common mutation with 72% higher frequency followed by codon 41/42 mutations with 11% lowest frequency [14]. A study on American blacks reported 17 different beta-thalassemia mutations, of which two are mild p+ thalassemia mutations (-88(C+T) and -29(A+G)) [15]. In the Turkish region, a rare mutation (IVS-I-130 (G-A)) was observed in beta-thalassemia major patients [16].

A study in Iranian populations revealed a 25 bp deletion, codon 30 (G>C), codons 36/37 (−T), and IVS-II-1 (G>A) as common mutations [17]. Awareness programs, carrier screening, and beta-thalassemia screening are crucial for reducing the prevalence of inherited diseases like beta-thalassemia, which impose significant burdens on families and society. Identifying mutations in the HBB gene through preliminary actions can help mitigate health disparities in vulnerable populations. This study focused on the exon 1 region of the HBB gene, unlike others that examined the entire gene, but did not explore intronic variants, which is a study limitation.

## Conclusions

Beta-thalassemia has posed significant challenges to affected families and the broader social community. Addressing the prevalence of such diseases has been imperative. Therefore, the establishment of genetic counseling and prenatal diagnosis for beta-thalassemia, leveraging mutational patterns, has shown promise in mitigating the disease burden. Screening all exons in the HBB gene has proven beneficial in preventing beta-thalassemia. Mutations have been observed to induce alterations in codon sequences, thereby affecting protein production. Analysis of mutations in exon 1 of the HBB gene has identified several common mutations, with missense mutations being predominant.
